# Cerebrospinal Fluid Biomarker Candidates for Parkinsonian Disorders

**DOI:** 10.3389/fneur.2012.00187

**Published:** 2013-01-21

**Authors:** Radu Constantinescu, Stefania Mondello

**Affiliations:** ^1^Department of Neurology, Institute of Neuroscience and Physiology, The Sahlgrenska Academy, University of GothenburgGothenburg, Sweden; ^2^Department of Anesthesiology, University of FloridaGainesville, FL, USA

**Keywords:** Parkinson disease, Parkinsonian disorders, cerebrospinal fluid, biomarkers, proteomics

## Abstract

The Parkinsonian disorders are a large group of neurodegenerative diseases including idiopathic Parkinson’s disease (PD) and atypical Parkinsonian disorders (APD), such as multiple system atrophy, progressive supranuclear palsy, corticobasal degeneration, and dementia with Lewy bodies. The etiology of these disorders is not known although it is considered to be a combination of genetic and environmental factors. One of the greatest obstacles for developing efficacious disease-modifying treatment strategies is the lack of biomarkers. Reliable biomarkers are needed for early and accurate diagnosis, to measure disease progression, and response to therapy. In this review several of the most promising cerebrospinal biomarker candidates are discussed. Alpha-synuclein seems to be intimately involved in the pathogenesis of synucleinopathies and its levels can be measured in the cerebrospinal fluid and in plasma. In a similar way, tau protein accumulation seems to be involved in the pathogenesis of tauopathies. Urate, a potent antioxidant, seems to be associated to the risk of developing PD and with its progression. Neurofilament light chain levels are increased in APD compared with PD and healthy controls. The new “omics” techniques are potent tools offering new insights in the patho-etiology of these disorders. Some of the difficulties encountered in developing biomarkers are discussed together with future perspectives.

## Parkinsonian Disorders

The Parkinsonian disorders have in common, to various degrees, the parkinsonism, defined as the presence of at least two of six movement abnormalities, of which either no. 1 or no. 2 are compulsory: (1) hypokinesia or diminished movement activity (also called bradykinesia, slowness of movement); (2) rest tremor; (3) rigidity (muscular stiffness); (4) loss of postural reflexes; (5) flexed posture; and (6) the freezing phenomenon (when the feet seem temporarily to be glued to the floor; Fahn, 2003). In addition to the motor abnormalities, specific combinations of non-motor symptoms such as autonomic and neuropsychiatric disorders, balance and ocular movement abnormalities, developing at various disease stages, characterize each particular Parkinsonian disorder, with major implications with regard to morbidity, treatment, and prognosis.

**Figure 1:**
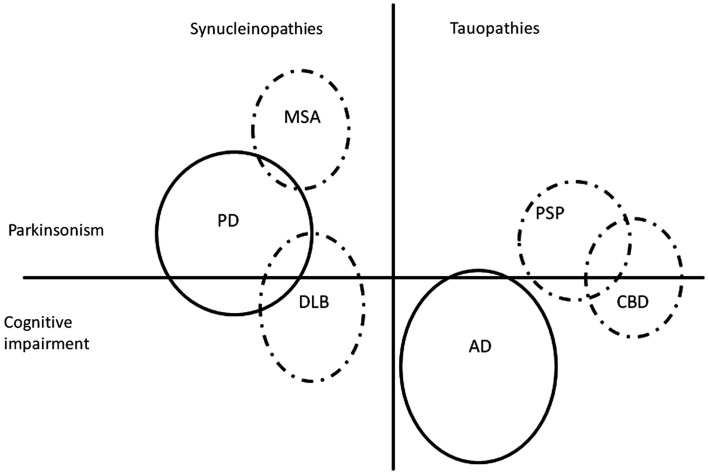
**Simplified and non-exhaustive visual representation of two groups of protein accumulation disorders (synucleinopathies and tauopathies), two major groups of symptoms (Parkinsonism and cognitive impairment), and some but not all possible interactions in-between**. All of the depicted disorders are Parkinsonian disorders with the exception of Alzheimer’s disease. The figure is not on scale. AD, Alzheimer’s disease; CBD, corticobasal degeneration; DLB, dementia with Lewy bodies; MSA, multiple system atrophy; PD, Parkinson’s disease; PSP, progressive supranuclear palsy. Dashed line, atypical Parkinsonian disorders.

The Parkinsonian disorders (Figure 1) represent a large group of neurodegenerative diseases affecting a considerable number of patients, most of whom are elderly. Parkinson’s disease (PD) dominates the group by far, as the most prevalent in the population, but also on scientific grounds, as a flagship for neurodegeneration in general, and due to the overwhelming impact which levodopa, its highly efficacious symptomatic treatment, has had on neurology. To the more uncommon atypical Parkinsonian disorders (APD) belong multiple system atrophy (MSA), progressive supranuclear palsy (PSP), corticobasal degeneration (CBD), and dementia with Lewy bodies (DLB). Depending on the nature of the abnormal proteins which aggregate in the nervous tissue in these diseases, they can be subclassified as either synucleinopathies (PD, MSA, and DLB) with alpha-synuclein accumulation, or tauopathies (PSP and CBD) with tau protein accumulation. The oftentimes deceptively similar clinical pictures of these diseases can make the differential diagnosis difficult, especially in early stages; generally, the clinical diagnostic accuracy is lower for APD compared with PD (Hughes et al., 2002). Due to the global aging of the population, the number of patients affected by these, for now, incurable disorders will expand in the future (Dorsey et al., 2007), with considerable strains on the health care system and society at large, increasing the need for developing new, efficacious therapies.

## Biomarkers

### Definition

The word “biomarker” is being used widely but not always correctly. The term was defined in 2001 by the Biomarkers Definitions Working Group as “A characteristic that is objectively measured and evaluated as an indicator of normal biological processes, pathogenic processes, or pharmacologic responses to a therapeutic intervention” (Biomarkers Definitions Working Group, 2001). Surrogate endpoints are a subgroup of biomarkers. They are a substitute for clinical endpoints which is what we really are interested in, reflecting how the patient is doing in reality. The requirements for a biomarker to serve as a surrogate endpoint are very strict and, at the present time, we do not have any surrogate endpoints in Parkinsonian disorders. However, any reliable biomarker, even if not strong enough to be a surrogate endpoint, would be tremendously valuable. In order for a parameter to be considered a biomarker for a certain disease, it must fulfill several requirements: (1) Validity: there must be a correlation between the biomarker and the disease which it stands for; a treatment must affect the disease and not only the biomarker itself; (2) Performance: how good is the biomarker? How well does it differentiate between affected and non-effected? The biomarker assessment must be reliable and reproducible, both in the same patient at different points in time, and at different centers. It must be feasible in a clinical context and that implies safety, tolerability, simplicity, and low cost; (3) Generalizability: the performance in different patient subsets, based, e.g., on age, gender, disease stage, and medication, must be known (Brooks et al., 2003; Marek et al., 2008). It is easy to use the word “biomarker,” but the implications of this word are profound, and despite all the efforts, we cannot say, for the time being, that we really have a biomarker for Parkinsonian disorders. What we do have in neurological sciences are: (1) biomarkers for certain disease-related processes, such as neurofilament light chain (NFL) as a biomarker of axonal degeneration, particularly damage to large-caliber, myelinated axons; and (2) different forms of protein inclusions, such as the 42 amino acid isoform of amyloid β (Aβ42) as a biomarker of Alzheimer-related senile plaque pathology.

### Types of potential biomarkers for parkinsonian disorders

There are different types of potential biomarkers for neurodegenerative disorders: biochemical analysis of blood, cerebrospinal fluid (CSF), urine or brain tissues, genetics, and multiple imaging modalities (e.g., different MRI techniques, SPECT, PET, and ultrasound of substantia nigra). In addition, several clinical markers are used to measure different aspects of the diseases and to track their progression: motor analysis; assessments of olfaction, autonomic functions, cognition, sleep, speech and swallowing, neuropsychological, and psychiatric investigations (Marek et al., 2008). This overview is only concerned with biochemical markers, mostly in the CSF but to a lesser degree also in the blood.

According to the aims of the investigation and the technique utilized, there are two main approaches to assess body fluids and body/brain tissues for biomarkers:

(1) Targeted search to investigate one or several *a priori* defined compounds in patients and in healthy controls and looking for differences, patterns, and associations.
(2) Untargeted search to investigate broadly a large amount of components in a sample and compare patients with healthy controls. Nowadays, this is achieved by the “omics” techniques.

## The “Omics” Techniques

The relatively new “omics” techniques present both an enormous potential, through their capacity of screening wide and complementary areas of different biological materials, and a significant challenge, through the huge amount of data that are generated and need interpretation. In biologic materials, transcriptomics, proteomics, and metabolomics evaluate the transient, momentaneous, or “state” characteristics of a sample while genomics mirror its permanent or “trait” characteristics.

### Genomics

Genomic studies survey and compare genomes in patients and controls, looking for associations between gene alleles, genetic risk factors, and disease. The more restricted candidate gene approach investigates specific genes in the context of a certain disease, such as mutations in the alpha-synuclein gene (SNCA) causing a rare form of autosomal dominant PD. The genome-wide association studies, a more recent technique, investigate the whole genome. Genetic studies and metaanalyses have found more than 16 PARK loci associated with PD and 11 genes for PARK loci, and new insights are gained every year (International Parkinson’s Disease Genomics Consortium and Wellcome Trust Case Control Consortium, 2011; Lill et al., 2012). Five of the identified genes induce a roughly typical PD presentation [a-synuclein, parkin, PTEN induced putative kinase 1, DJ-1, and leucine-rich repeat kinase 2 (LRRK2)] while mutations of ATP13A2 (PARK9) cause Kufor–Rakeb disease characterized by both Parkinsonism and many atypical features (Coppede, 2012). A genetic biomarker is unchangeable and indicates a trait, a predisposition to develop a disease. However, it does not indicate whether the disease has started or how advanced it is; it does not provide information about the state. Due to environmental factors, age, or reduced penetrance, the trait may or may not induce a state of disease during the lifetime of the bearer. The LRRK2 mutation is an example of a genetic trait for an autosomal dominant form of PD with variable penetrance probably due to non-genetic factors. Through genome-wide association studies, Simon-Sanchez et al. (2009) found a strong association in PD with the alpha-synuclein gene (SNCA) and, surprisingly for a synucleinopathy, also with the MAPT locus, related to tau protein.

An emerging research field is epigenetics which may bridge the gap between the apparently unchanging genome and the ever changing environment. There is evidence from both human but mostly from *in vitro* and animal models that DNA methylation, histone modifications, and small RNA-mediated mechanisms, could modify the expression of PD-related genes such as the alpha-synuclein gene, DJ-1, LRRK2, and parkin-gene, and thereby contributing to the development of the disease (Marques et al., 2011; Coppede, 2012).

### Transcriptomics

Transcriptomics investigates mRNA levels of expressed genes coding for proteins. Several studies have examined cells from substantia nigra in PD patients, controls, and PD animal models. Differences were found between controls and patients but the results in regard to particular genes were not similar between studies (Smith, 2009; Caudle et al., 2010). However, looking at patterns, findings became more consistent across studies and a pattern could be discerned showing that genes involved in oxidative stress, mitochondrial function, protein degradation, dopaminergic transmission, and axonal guiding were expressed differently in the different diagnostic groups (Smith, 2009; Caudle et al., 2010).

### Proteomics

Proteomics characterizes the protein content – the proteome of a sample. Comparing the proteomes of patients and controls, differences may be found. The technology is based on three components: (1) separation of proteins; (2) analyzing proteins through mass spectrometry; and (3) quantifying and identifying the proteins through advanced data processing (Caudle et al., 2010). Using this technique, a comprehensive characterization of the proteome in substantia nigra was made by one group (Kitsou et al., 2008). Many of the proteins known to be involved in PD such as DJ-1 and UCHL-1 were identified. Using proteomics, the proteome of the CSF was characterized and over 1500 proteins were identified and grouped according to their functions, such as cell cycle, signal transduction, and cellular transport. In addition, a large number of proteins unique to PD, AD, and DLB were identified (Abdi et al., 2006). Seventy two of them were uniquely altered in PD compared with healthy controls. Apolipoprotein H (Apo H) and ceruloplasmin appeared to be able to segregate PD from healthy controls and from non-PD (AD and DLB). Using the same material, Zhang et al. (2008) validated a multianalyte CSF profile, identifying a panel of eight CSF proteins that were highly effective at recognizing PD. In a study in PD, MSA, CBD, PSP, and healthy controls, a panel of four proteins (ubiquitin, β2-microglobulin, and 2 secretogranin 1 [chromogranin B] fragments) was identified which could differentiate PD and healthy controls on one side from APD on the other side with an AUC of 0.8 (Constantinescu et al., 2010a).

Subcellular proteomics investigates the proteome at the subcellular level, in compartments of the cell. Such a compartment is neuromelanin, a granular pigment associated with lysosomes and present in cathecolaminergic neurons. It interacts with compounds in the cytoplasm such as iron, lipids, pesticides, neurotoxins, and it sequesters them, thus having a cytoprotective function. However, if it malfunctions, it could turn out to become cytotoxic and be involved in neurodegeneration. The proteins associated with neuromelanin were investigated using proteomics (Tribl et al., 2006). Several were associated with mitochondrial function and chaperons. Interestingly, antibodies against neuromelanin have been found in serum from PD patients (Double et al., 2009). Subcellular proteomics was also used for analyzing Lewy bodies. Several proteins thought to be involved in the pathogenesis of PD were found, associated with alpha-synuclein, such as chaperons, proteins involved in oxidative stress, and proteosomal degradation (Xia et al., 2008). Analyzing mitochondrial fractions, 119 proteins were found to differ in PD compared with controls. Especially interesting is mortalin, involved in mitochondrial function and oxidative stress reactions. Low levels of mortalin were found in substantia nigra from PD patients compared with controls (Jin et al., 2006).

A shortcoming of the proteomics technique is that it is often biased toward identification of abundant proteins. As albumin and immunoglobulins represent more than 70% of CSF proteins, a way to enhance the discovery of proteins present in small amounts is to exclude the abundant proteins from the sample through fractionation. Blood contamination with its high protein content can dramatically alter CSF proteomic pattern and it has been suggested to exclude from proteomic analyses CSF containing more than 10 erythrocytes per microliter (Caudle et al., 2010).

### Metabolomics

Metabolomics investigates end products of metabolic pathways. These are molecules with low molecular weights required for the maintenance, growth, and normal function of a cell (Beecher, 2003). Adequate sample collection and preparation prior to analysis is very important for accurate results. Metabolomic studies conducted by Bogdanov et al. have confirmed the inverse association between blood urate levels and the risk for PD. In addition, they found higher levels of glutathione and 8-hydroxydeoxyguanosine (8-OHdG) in PD compared with controls. These compounds are markers of oxidative processes and support the oxidative stress hypothesis in PD (Bogdanov et al., 2008). The same group could differentiate controls from idiopathic PD patients, patients with idiopathic PD from those with hereditary PD caused by the G2019S variant of the LRRK2 mutation, and also symptomatic LRRK2 mutation carriers from asymptomatic carriers, based on the metabolomic profile (Johansen et al., 2009).

### Conclusion

Ideally, findings from the four “omics” techniques applied on different materials (e.g., substantia nigra cells or the CSF) should be consistent. Thus, if genomics shows an altered gene in neuronal nuclei, then the mRNA (transcriptomics) should reflect that in the cytoplasm, and further, after translation, in proteins and through them metabolic products detected in the cell or in the CSF by proteomics and ultimately by metabolomics. Findings in the CSF should be replicated in substantia nigra cells. Unfortunately, this congruence of findings is not often to be seen. That may be due to the limitations of the techniques or experimental incongruences, along with the use of different techniques and the inherent complexities of living organisms (Caudle et al., 2010). Better equivalence is achieved when findings from different techniques are categorized within pathways such as oxidation, synaptic transmission, mitochondrial function, or protein degradation. Of these, the oxidative stress pathway is the most robust with similar results from both cellular and CSF analysis, from genomics, transcriptomics, proteomics, and metabolomics. Thus, oxidative stress appears to be the final common pathway in the neurodegenerative process in PD (Caudle et al., 2010). Better integration of these techniques should lead to a deeper understanding of the pathophysiology of PD as well as other neurodegenerative disorders, and open venues for developing new treatment strategies.

## Cerebrospinal Fluid

The first lumbar puncture (LP) was done in London 1889 and CSF studies have a long tradition in neurology, both in research and in clinical practice (Frederiks and Koehler, 1997). We know mainly from AD research that CSF studies in patients with neurodegenerative disorders are feasible with a low rate of post LP headache or other complications (Andreasen et al., 2001) and CSF analysis for assessing tau protein and beta-amyloid belongs now to the standard of care in the management of dementias. Brain-derived proteins do not usually appear in the blood due to the blood-brain barrier. In contrast, CSF is very close to the pathologic processes in the brain, and may better reflect changes in brain metabolism (Mollenhauer and Zhang, 2012). This may offer advantages when investigating neurodegenerative disorders. Even though protected by the blood-brain barrier, the CSF is dynamic. Proteins that diffuse in the CSF from plasma have a concentration gradient with a 2.5 times higher lumbar concentration than cranial. Proteins secreted in the CSF from the brain have about the same concentration in the CSF space, but some, including tau protein, may actually have a lower concentration distally, in the lumbar region. There are also diurnal variations, as the secretion of proteins into the CSF is higher at night. In addition, the protein concentration decreases between the first ml CSF tapped at the LP and the later portion which is the preferred one as it more accurately reflects the environment in the brain. All this makes imperative the standardization of the CSF sampling protocol (Kroksveen et al., 2011).

It has been suggested that CSF itself mediates humoral signaling which is distinct from synaptic neurotransmission. In one study, spherical nanometric-scale structures were identified in the CSF containing synaptic vesicles (Harrington et al., 2009). Cell-line studies have shown that CSF from PD patients affects dopaminergic cells differently than CSF from healthy controls, implying that there are differences in their composition (Le et al., 1999). Due to all this, CSF has been widely investigated in Parkinsonian disorders and it might be considered to offer the most promising insights in the disease processes (Lewitt, 2012).

There have been concerns regarding CSF sample handling and its impact on the acuity of CSF data as post-translational modifications, protein loss, and degradation can be caused by non-optimal CSF related procedures including sampling, freezing, thawing, and storage. Therefore it is important to have standard operating procedures in place (Lewczuk et al., 2006). A consensus protocol for the standardization of CSF collection and handling has been published in 2009 and is being followed by many European centers (Teunissen et al., 2009). In regard to analysis, for increasing the reliability of results, a study should ideally include a training subgroup and a validation subgroup, the latter preferably run by a different research group (Zetterberg et al., 2008; Mollenhauer and Trenkwalder, 2009).

## CSF Biomarker Candidates for Parkinsonian Disorders

In a review by Mollenhauer et al. from 2008 of all then current publications regarding CSF biomarkers in PD, MSA, PSP, CBD, and DLB, no less than 67 tested compounds were identified, most of them in PD. However, several limitations were found in most of the studies: sensitivity and specificity were low; there was a lack of reproducibility of results by independent cohorts; and the analysis methods in use were still considered to be in their infancy (Mollenhauer and Trenkwalder, 2009). Thus, there is no scarcity of investigations on CSF compounds with biomarker potential in Parkinsonian disorders. What we barely have are mature CSF biomarker candidates and what we still lack is a real biomarker.

Historically, due to the prominence of the dopaminergic abnormalities in these disorders, the first compounds to be tested were dopamine and other monoamines and their metabolites. As these results were prone to be influenced by a multitude of other factors, the quest went further to compounds which were already known and tested in other diseases such as tau protein, beta-amyloid, and NFL. With advancing knowledge and technical capabilities, the search turned further toward specific targets following theoretical considerations in regard to patho-etiology, such as alpha-synuclein, or inflammatory markers. Later on, the newer and far-reaching possibilities offered by the “omics” techniques led to broad searches surveying large, nod-discriminate entities like the genome or the proteome. The overview presented here has no claim on being exhaustive; instead it focuses on a number of compounds perceived to be more mature and/or promising for the future.

### Specific biomarker candidates in the CSF and blood

A summary is presented in Table 1.

**Table 1:** **Cerebrospinal fluid biomarker candidates in Parkinsonian disorders**.

Compound	PD	MSA	PSP	CBD	Conclusion
Alpha-synuclein	↓ ↔	↓ ↔	↔ ↑	↔	Decreased in PD and MSA but not in PSP and CBD. Inconsistent data
NFL	↔	↑	↑	↑	NFL normal in PD but increased in MSA, PSP, and CBD, vs. controls
Total tau protein	↓ (↑) ↔	↑ ↓ ↔	↔	↑ ↔	Decreased in PD and increased in CBD. Inconsistent data
Aβ42	↓ ↔	↓ ↔	↓ ↔	↓ ↔	Decreased in PDD and DLB. Inconsistent data in PD, MSA, PSP, and CBD
DJ-1	↑ ↓	–	–	–	Data is not consistent
8-OHdG	↑	–	–	–	Limited results. Probably increased in PD
Urate	[↓]	[↓]		–	Lower urate levels are associated with a higher risk for developing PD and with a faster rate of disease progression in PD and MSA

*Aβ42, amyloid-β; CBD, corticobasal degeneration; DLB, dementia with Lewy bodies; MSA, multiple system atrophy; NFL, neurofilament light chain; PD, Parkinson’s disease; PDD, Parkinson’s disease with dementia, PSP, progressive supranuclear palsy; vs., versus. 8-OHdG, 8-hydroxydeoxyguanosine*.

#### Alpha-synuclein

##### Background

Alpha-synuclein is the main component of intracytoplasmatic Lewy bodies and of Lewy neurites in neuronal processes. These structures are found in PD and in DLB in the remaining dopaminergic neurons in substantia nigra, and also in non-dopaminergic cortical and non-cortical neurons (Jellinger, 1990, 2003). In MSA, alpha-synuclein is a component of the characteristic glial intracytoplasmatic inclusions.

Mutations affecting the gene coding for alpha-synuclein cause rare hereditary forms of PD, such as in PARK1 (missense) and PARK4 (duplication, triplication; Polymeropoulos et al., 1997) but are also important for sporadic forms of PD (Farrer et al., 2001). In addition, in both PD and MSA, genome-wide association studies showed a strong association between disease risk and distinct single nucleotide polymorphisms (SNPs) in the α-synuclein encoding gene (Simon-Sanchez et al., 2009). There seems to be a dose-effect of alpha-synuclein as increased levels of synuclein caused by duplications and triplications of the gene cause PD (Fuchs et al., 2008; Simon-Sanchez et al., 2009).

##### Alpha-synuclein’s role in the pathogenesis of synucleinopathies

Although it is widely expressed in the brain, the precise function of alpha-synuclein is not known. It might play an important role in neurotransmission by regulating synaptic vesicle size and recycling. Mutant alpha-synuclein builds fibrils, aggregates, resists degradation, and ultimately interferes with vital cell functions such as transcription, the ubiquitin-proteasome system, lysosomes and mitochondria, disrupting protein metabolism, and energy production. Oxidation, pesticides, and mitochondrial dysfunction can damage alpha-synuclein and initiate its metamorphosis to toxic forms (Moore et al., 2005). It has been proposed that alpha-synuclein pathology and subsequent neurodegeneration could represent a common event for different forms of PD, with different etiologies. A recent theory proposes pathologic “seeding” throughout the nervous system of abnormal alpha-synuclein which, after finding its way in the body, might, through a prion-like induction, spread from cell to cell, causing the neurodegenerative process in PD (Angot et al., 2010; Jucker and Walker, 2011). Due to alpha-synuclein’s prominence in the pathogenesis of these disorders, PD, MSA, and DLB are considered to be synucleinopathies.

##### Previous findings in Parkinsonism

Cerebrospinal fluid alpha-synuclein levels in PD have been investigated using different techniques in over 10 studies. A majority of them showed decreased levels in PD (Tokuda et al., 2006; Mollenhauer et al., 2008; Hong et al., 2010; Mollenhauer et al., 2011) but not all (Borghi et al., 2000; Ohrfelt et al., 2009).

Four studies have investigated CSF alpha-synuclein levels in MSA. Three of them found decreased levels in MSA compared with controls but not with PD patients (Mollenhauer et al., 2011; Shi et al., 2011; Hall et al., 2012). In one of them levels were similar in MSA, PD, and controls (Tateno et al., 2012). In one study, PD and MSA could be differentiated by the CSF Flt3 ligand, not by alpha-synuclein (Shi et al., 2011).

In one study, CSF alpha-synuclein levels in PSP and CBD were not significantly different compared with controls. However, levels in PSP but not in CBD were higher than in PD (Hall et al., 2012).

Alpha-synuclein levels have also been investigated in plasma in PD and MSA but with conflicting results. Both higher (Lee et al., 2006) and similar (Li et al., 2002) levels compared with controls have been found and there was no correlation with PD severity. A major difficulty in measuring both alpha-synuclein and DJ-1 in plasma is the risk for contamination with erythrocytes or platelets as more than 95% of these compounds reside in erythrocytes and about 4% in platelets. However, even after controlling for that, there were no statistically significant differences between PD patients and controls in regard to these compounds although there was a trend for lower levels in PD. It does not seem that plasma alpha-synuclein can be used as a biomarker for PD for the time being (Shi et al., 2010).

Oligomeric forms of alpha-synuclein protein in plasma were higher in PD than in controls, in one study (El-Agnaf et al., 2006). However, in another study, phosphorylated alpha-synuclein, but not total alpha-synuclein nor oligomers of alpha-synuclein, was higher in PD than in controls (Foulds et al., 2011). Interestingly, antibodies directed against monomeric alpha-synuclein were found in plasma of PD patients, with higher response in earlier disease phases (Yanamandra et al., 2011). Studies in animal models suggest that immunomodulatory interventions such as vaccination with alpha-synuclein (Masliah et al., 2005) or administration of alpha-synuclein antibodies (Masliah et al., 2011) may have a positive impact on the intraneuronal accumulation of alpha-synuclein, presumably reflected by reduced neuropathological and behavioral deficits. Intravenous immunoglobulin reduced alpha-synuclein oligomer neurotoxicity in human neuroblastoma cells (Smith et al., 2012). These results may motivate further research aiming to find whether immunomodulation might be a novel therapeutic approach in PD.

Alpha-synuclein was found not only in the brain and the blood but in other peripheral locations too. It was found in the colonic mucosa years before the emergence of PD symptoms and the question was raised whether it can be a biomarker for premotor PD stages (Shannon et al., 2012a,b). In saliva, alpha-synuclein was lower in PD patients than in controls and it inversely correlated with the UPDRS score (Devic et al., 2011).

Cerebrospinal fluid alpha-synuclein levels increase non-specifically in Creutzfeldt–Jakob’s disease, presumably due to massive neuronal death (Mollenhauer et al., 2008). The same phenomenon but on a smaller scale occurs in AD, with increased CSF alpha-synuclein levels (Hall et al., 2012).

Although alpha-synuclein is a strong biomarker candidate due to its important role in the pathogenesis of synucleinopathies and to several promising results, currently it cannot be considered a mature biomarker. However, in a group of parkinsonian patients, low CSF alpha-synuclein levels could help with their stratification, due to its high positive predictive value for synucleinopathies. An additional marker (e.g., non-motor prodromal symptoms) would strengthen the stratification process and help to select a group of patients who may benefit from future synuclein-reducing therapies (Mollenhauer et al., 2011). Longitudinal studies and studies in early disease stages are needed in order to better understand the value of alpha-synuclein as potential biomarker in Parkinsonism.

#### Neurofilament light chain protein

##### Background

Neurofilaments (NF) are major neuronal structural elements, composing the intermediate filaments present in nerve fibers. They are mainly involved in maintaining the axonal caliber and the neuronal shape and size (Lasec, 1988) and are thereby critical for the morphological integrity of neurons and for the conduction of nerve impulses along the axons (Hoffman et al., 1987). The NF are composed of three subunits of different molecular weights: light chain NF (NFL), medium chain NF (NFM), and heavy chain NF (NFH). The NFL forms the backbone to which NFH and NFM chains copolymerize to form NF. Increased levels of CSF NF primarily reflect axonal degeneration of large myelinated axons, such as those present in the pyramidal tracts. NFL is a mainly non-phosphorylated protein, whereas NFH is substantiality phosphorylated (pNFH), and can be measured in that form. CSF NFL has been shown to be increased in a variety of acute and chronic neurological diseases (Rosengren et al., 1996; Rosengren et al., 1999; Zetterberg et al., 2006; for review, see Norgren et al., 2003).

##### Previous findings in Parkinsonism

NFL has been investigated in Parkinsonian disorders in a relatively large number of studies (Holmberg et al., 1998; Holmberg et al., 2001; Abdo et al., 2007a; Abdo et al., 2007b). A review from 2009 concluded that NFL could differentiate between PD and controls on one side and MSA and PSP on the other side, although with overlap. NFL could not discriminate between MSA with predominant Parkinsonism and MSA with predominantly cerebellar symptoms, nor between MSA and PSP (Constantinescu et al., 2009). Consecutive analyses of CSF NFL did not show any significant changes over 1 year and no correlation with disease severity. CSF NFL levels were also increased in CBD (Constantinescu et al., 2010b). Several studies have been conducted since then with similar findings (see Combinations of CSF Compounds for the most recent results). Hall et al. (2012) found increased NFL in MSA, PSP, and CBD. In one study in advanced PD patients treated with deep brain stimulation of nucleus subthalamicus, CSF NFL levels increased sharply directly after surgery but normalized gradually and were normal at 1 year and later. Thus, using this method, no signs of accelerated neuronal death due to active DBS could be found (Constantinescu et al., 2011). To be able to ascertain that a therapy is not in itself deleterious for the disease being treated remains a key point, and even more as new therapeutic approaches to PD are envisioned that employ potentially harmful techniques (e.g., intracranial catheters for injection of neurotrophic factors, cell transplants, and genetical modifications using viral vectors). Thus, in the future there may arise the need to detect adverse events using a sensitive, albeit non-specific, marker for brain damage. In this context, CSF NFL with its high sensitivity for detecting more aggressive neuronal death than it occurs in PD, even if enfeebled by a low diagnostic specificity, might be of use.

#### Tau protein

##### Background

Tau protein is important for the function of axonal microtubules and thereby for the structural integrity of the neuron and for axonal transport. In hyperphosphorylated form it has reduced binding affinity for microtubules and leads to their malfunction. At the same time, it adopts an abnormal configuration favoring aggregation and inclusion formation (Kouri et al., 2011). Tau protein is the main structural element of neurofibrils in Alzheimer’s disease (AD) but it has also been found in neurofibrillary tangles in PSP, in neuronal cytoplasmatic inclusions, and in ballooned neurons in CBD and PSP (Mori et al., 1994).

##### Previous findings in Parkinsonism

Cerebrospinal fluid tau protein levels in Parkinsonism have been investigated in many studies in the past, with inconclusive results. In PD, most studies found normal values, but both higher and lower values were reported. In atypical Parkinsonism, tau levels tended to be higher in MSA than in PD, but not in PSP. The results for CBD are mixed, with both higher and lower levels than in controls being reported (for review of older literature, see Constantinescu et al., 2009).

Recently, in a large study on patients with dementia, total tau and phosphorylated tau levels were not significantly different in PSP and CBD compared with controls (patients with subjective memory complaints; Schoonenboom et al., 2012). In four recent large studies, tau protein was investigated along with other CSF compounds (see Combinations of CSF Compounds).

#### Amyloid-β

##### Background

Aβ42, derived from the proteolytic processing of a larger protein, amyloid precursor protein, is a major component of neuritic plaques in AD. Due to its sequestration in plaques, the characteristic pattern in AD is low CSF Aβ42 levels. Low CSF concentrations have also been found in Creutzfeldt–Jakob’s disease, in DLB, in frontotemporal and vascular dementias, and in PD with dementia.

##### Previous findings in Parkinsonism

Previous studies in Parkinsonism were inconclusive, with both normal and decreased levels in the same disorder, and did not allow drawing any conclusions (Hall et al., 2012; for review, see Constantinescu et al., 2009). However, *in vitro* studies have shown that Aβ42 promotes accumulation of alpha-synuclein making it interesting in a PD context (Masliah et al., 2001).

More recent studies have found a correlation between Aβ42 and cognitive dysfunction in PD, with significantly lower CSF Aβ42 and higher total tau protein levels in Parkinson’s disease with dementia (PDD) compared with PD (Mollenhauer et al., 2006). In addition, this pattern also distinguished AD from PD, DLB, and MSA, although CSF Aβ42 was lower in DLB compared with controls and PD (Zhang et al., 2008; Shi et al., 2011; Hall et al., 2012). In a study from Norway, non-demented PD patients with memory impairment had lower Aβ42 than those without memory impairment (Alves et al., 2010). Significant associations were found between cognitive performance and CSF levels of Aβ42 and Aβ42/total tau (Leverenz et al., 2011). Interestingly, in a rare occurrence, the ratio fractalkine/Aβ42 correlated with PD severity assessed by UPDRS-III (Shi et al., 2011).

#### DJ-1

##### Background

DJ-1 is a gene product associated with PD in both familial and sporadic forms. Its exact function is not known but it seems to play an important role in oxidative processes where it probably acts as a protease, chaperon, or antioxidant (Choi et al., 2006). Loss of DJ-1 function leads to neurodegeneration.

##### Previous findings in Parkinson’s disease

Previous studies have found both higher (Waragai et al., 2006) and lower (Hong et al., 2010) CSF DJ-1 levels in sporadic PD compared with non-PD controls. DJ-1 will be investigated in the ongoing Parkinson Progression Markers Initiative study aiming to identify markers for disease progression (Parkinson Progression Marker Initiative, 2011).

#### 8-Hydroxydeoxyguanosine

##### Background

8-Hydroxydeoxyguanosine (8-OHdG) is produced when reactive oxygen radicals react with guanine residues in DNA. When the oxidized DNA is repaired, 8-OHdG is excreted in the blood and eventually in urine, where it can be measured. As such, it has emerged as a marker of oxidation and mitochondrial dysfunction, not only in neurodegenerative disorders but also in cancer research.

##### Previous findings in Parkinson’s disease

Sato et al. (2005) found that the mean urinary 8-OHdG increased with the disease stage in PD patients and another group found an association between hallucinosis in PD and urinary 8-OHdG levels (Hirayama et al., 2011).

The CSF 8-OHdG levels were increased in non-demented PD compared with controls (Gmitterova et al., 2009). 8-OHdG is one of the parameters selected for assessment in the FS-ZONE study, investigating the effect of pioglitazone, a potential antioxidant, in early PD^1^. Increased 8-OHdG blood levels in PD were identified in metabolomic studies as previously discussed as previously discussed in the metabolomics Section 4.1.4.

#### Urate

##### Background

In humans, uric acid is the major product of the catabolism of the purine nucleosides adenosine and guanosine. Purines are derived from dietary intake as well as from endogenous metabolic processes (synthesis and cell turnover). The enzyme uricase which breaks down urate is absent in humans and apes, due to mutations which occurred millions of years ago (Wu et al., 1989). As a result, along with an extensive reabsorption of filtered urate (>90%), humans have high serum urate levels (about 5 mg/dL in men), close to the maximum solubility. Levels above the saturation limit (7 mg/dL) can result in hyperuricemia which may be a cause of disease in humans. However, higher urate levels may account for the greater longevity of humans, e.g., due to lower cancer rates compared with shorted-lived mammals. During the evolution, urate has replaced ascorbate as the most potent antioxidant in humans.

##### Previous findings in Parkinsonian disorders

There is a substantial amount of evidence showing a relationship between urate and PD. Higher serum urate levels and higher dietary urate intake are associated with lower risk for developing PD, and with slower disease progression, better cognitive performance, and reduced loss of striatal [123I] β-CIT uptake in patients already having PD (Davis et al., 1996; Annanmaki et al., 2007; Annanmaki et al., 2008; Ascherio et al., 2009). In a recent study, the ratio between the immediate precursor of urate, xanthine, and homovanillic acid, the major catabolite of dopamine, was different in PD patients compared with controls and correlated with disease severity (Lewitt et al., 2011). The odds for having parkinsonism but without signs of dopaminergic deficit on iodine-123-labeled 2-β-carboxymethoxy-3-β-(4-iodophenyl) tropane ([123I]b-CIT) scan were higher in subjects with higher urate levels (Schwarzschild et al., 2011). In one small study serum urate levels were higher in tauopathies compared with synucleinopathies (Constantinescu et al., 2012). In MSA, higher serum urate was associated with a lower rate of disease progression (Lee et al., 2011). In DLB, serum urate levels were lower than in controls (Maetzler et al., 2011). There are discrepancies in the reported data concerning the importance of gender in this context. Some studies have found the association with urate levels to be significant in men only, others in both genders.

The title of a recent article reflects the encouraging data centered on urate and its future perspectives: “Urate: a novel biomarker of PD risk, diagnosis, and prognosis” (Cipriani et al., 2010).

#### Peroxisome proliferator-activated receptor gamma coactivator-1 alpha

##### Background

Peroxisome proliferator-activated receptor gamma coactivator-1 alpha (PGC-1α) is a key transcriptional co-regulator involved in mitochondrial respiration, oxidative stress defense, and adaptive thermogenesis (Puigserver and Spiegelman, 2003).

##### Previous findings in Parkinson’s disease

Reduced mRNA levels of PGC-1α leading to mitochondrial dysfunction and neurodegeneration were found in Huntington’s disease models (Cui et al., 2006), opening up for new therapeutic targets (McGill and Beal, 2006). The same phenomenon seems to occur in PD (Keeney et al., 2009; Pacelli et al., 2011) and PGC-1α is under investigation in new PD studies such as Pioglitazone in Early PD^2^ (FS-ZONE).

#### Combinations of CSF compounds

Hong et al. investigated PD patients, healthy controls, and AD patients, and found that both DJ-1 and alpha-synuclein were decreased in PD compared with the other groups. Alpha-synuclein discriminated PD from controls with a sensitivity of 92% and a specificity of 58%. For DJ-1 the sensitivity was 90% and the specificity 70%. There was no association with disease severity. Combining alpha-synuclein with DJ-1 did not enhance the performance of the test model. They emphasized that blood contamination must be an exclusion criterion for sample analysis as it influenced the results; likewise, age must be taken into consideration as both DJ-1 and alpha-synuclein increased with age (Hong et al., 2010).

Mollenhauer et al. investigated a large number of patients with both synucleinopathies (PD, MSA, and DLB) and tauopathies (PSP and AD) plus neurological controls, first in a training set and afterward in a validation set. They found that a CSF alpha-synuclein concentration of 1.6 pg/μL discriminated PD from non-synucleinopathies with a 70% sensitivity and a 53% specificity. At this cut-off, the positive predictive value for any synucleinopathy was 91%. In the training set, a combination of alpha-synuclein, tau protein, and age discriminated between synucleinopathies and neurological controls and AD with an area under the curve (AUC) of 0.908. In the validation cohort the AUC was 0.702 for discriminating between synucleinopathies and a mixture of PSP, normal pressure hydrocephalus, and neurological controls. Age, not diagnosis, was the strongest factor affecting total tau protein levels. Only mean alpha-synuclein levels and not total tau, or Aβ42 levels differentiated PD and MSA from neurological controls (Mollenhauer et al., 2011).

Hall et al. assessed patients with synucleinopathies (PD, MSA, DLB, and PDD), tauopathies (PSP, CBD, and AD) and healthy controls using a panel of compounds: alpha-synuclein, total tau protein, hyperphosphorylated tau, Aβ42, and NFL. Alpha-synuclein levels were decreased in synucleinopathies compared with controls, PSP, and AD. NFL levels were substantially increased in APD. A receiver operating characteristics (ROC) analysis conducted to determine the value of NFL to differentiate PD from APD resulted in an AUC of 0.93. Total tau protein was decreased in PD compared with controls, but increased in MSA and CBD compared with PD. No significant change was seen in PSP. Aβ42 did not differ significantly between controls and PD, MSA, PSP, and CBD (Hall et al., 2012).

Shi et al. examined patients with PD, MSA, AD, and healthy controls. The fractalkine/Aβ1–42 ratio correlated positively with PD severity (in cross-sectional studies) and with PD progression (in longitudinal studies). No other marker had shown this association before. Fractalkine is important for the proper function of microglia. In addition, the Flt3 ligand, a cytokine which acts as a neurotrophic and anti-apoptotic factor in CNS, could alone differentiate between PD and MSA with a sensitivity of 99% and a specificity of 95%. Aβ1–42 levels were lower in PD and MSA than in controls but higher than in AD. They could not differentiate between PD and MSA. Total tau levels were also lower in PD and MSA than in controls and AD. A combination of alpha-synuclein and phosphorylated tau/total tau could also differentiate PD from MSA with a sensitivity of 90% and a specificity of 71% but only when samples with blood contamination were excluded. Alpha-synuclein was decreased in both PD and especially in MSA compared with controls, presumably reflecting aggregation or metabolic abnormalities (Shi et al., 2011).

Bech et al. investigated a group of patients with Parkinsonian disorders (PD, MSA, PSP, CBD, DLB, and PDD). They could confirm previous results concerning NFL. Thus, a ROC analysis of NFL showed a sensitivity of 86% and a specificity of 81% with a cut-off value of 284.7 ng/L for differentiating PD from atypical parkinsonism. Aβ42 was low in DLB. Neither phosphorylated tau nor total tau differed between the diagnostic groups (Bech et al., 2012).

## Why are Biomarkers for Parkinsonian Disorders Needed?

The ultimate reason for needing a biomarker is the fact that we still do not have any disease-modifying treatment in movement disorders. The lack of biomarkers is considered to be one of the greatest limitations for developing such a treatment (Olanow et al., 2008). Over years, there has been no shortage of therapeutic hypotheses or compounds to be tested; the list with failed compounds is very long. The real problem has been the lack of a reliable way to assess the underlying disease process and whether an intervention could influence it and alter the course of the disease (Ravina et al., 2003; Kieburtz and Ravina, 2007; Sherer, 2011).

It has been assessed that it takes 5 years of follow-up and 600 subjects participating in a randomized placebo-controlled trial in order to detect a 20% slowing of functional decline. A biomarker could dramatically reduce the resources needed for that (Hersch and Rosas, 2011).

Considering the very nature of Parkinsonian disorders and the limits it puts on the process of developing disease-modifying therapies, biomarkers could be useful for solving many limiting issues.

### The differential diagnostic issue

Differential diagnosis can be difficult during early phases of Parkinsonian disorders. What might look as PD in the beginning could turn out to be PSP, MSA, or even CBD. What was initially considered to be a synucleinopathy may end as a tauopathy. Ultimately, the gold standard for diagnosis remains neuropathology. Considering the substantial differences between these disorders, mixing together patients with different diagnoses may lead to negative or inconclusive results in any therapeutic trial, even when the therapy itself is efficient for one of these diagnoses. A biomarker pointing early toward the right diagnosis would increase the probability of success.

A diagnostic biomarker would decrease the cost, time, and effort it would take to secure a diagnosis. Currently, that is best achieved through an assessment done by a movement disorders specialist. A biomarker would simplify the diagnostic process.

Even when there is no doubt regarding diagnosis, an ideal biomarker could help stratify patients in subgroups which may show different responses to a given therapy. That would make possible a distinction between respondent and non-respondent diagnostic subgroups, preventing the dismissal of a therapy when it does not benefit the diagnostic group as a whole. Such a distinction would also permit, within a given diagnostic group, to differentiate and individualize treatment according to expected benefits or risks, and expected disease progression and complications (Marek et al., 2008). For example, young PD patients with an increased risk for developing dyskinesias, once levodopa therapy is instituted, might need a different treatment approach compared with patients with late disease onset and a low risk for dyskinesia but high for dementia.

### The time of disease onset and progression issue

To date, it is impossible to determine the exact date of onset in Parkinsonian disorders. Once started, the disease is asymptomatic for several years, followed by the emergence of non-specific, non-diagnostic symptoms. Our “early” diagnosis based on the emergence of motor symptoms probably describes an already advanced disease process.

Thus, in PD, it has been calculated that up to 50–70% of substantia nigra neurons are lost before symptomatic motor abnormalities develop (Fearnley and Lees, 1991) and the premotor period could be between 5 and 20 years long (Marek et al., 2008). In one positron emission tomography study in PD, a mean preclinical period of 5.6 ± 3.2 years was calculated (Hilker et al., 2005). Results from the Honolulu-Asia Aging Study do also place the onset of non-motor symptoms, such as bowel movement abnormalities, 10 years or more before the emergence of diagnostic motor symptoms (Abbott et al., 2001).

The fact that the disease onset predates with years the time when enough symptoms emerge for a diagnosis to be made, implies that even efficacious therapies may show themselves powerless if given when neurodegeneration has gone that far (Stern et al., 2012). An ideal biomarker could detect the disease in presymptomatic individuals or early in the disease course allowing an efficacious disease-modifying therapy to act and “cure” or at least delay the progression of disease.

For now, there is also no way of measuring disease progression. The tools we have been using are clinical scales of which UPDRS (Fahn et al., 1987) is the most widespread for PD and the Unified Multiple System Atrophy Rating Scale (UMSARS) for MSA. However, these scales are no biomarkers and they are subject to both investigator and patient bias and cannot be considered truly objective; they are not reliable as their score can vary from hour to hour due to medication, placebo, food intake, or a myriad of other causes; they measure a combination of dopaminergic and non-dopaminergic effects and not the disease process itself, nor the direct effects of treatment over this process. Biomarkers are needed to identify the development of disease, and monitor and measure its progression.

### The effects of therapy issue

At the present time we do not have a way of assessing whether and to which degree a therapeutic intervention has an impact on the disease process: we cannot measure the effects of a therapy. The clinical scales which we use today are subject to error, as discussed before. In addition, as it was shown in the ELLDOPA study, clinical measures such as UPDRS, and a more objective assessment, radiotracer imaging, moved in different directions after the therapeutic intervention, levodopa treatment, leading to confusion in regard to interpretation (Fahn et al., 2004). A further problem is that radiotracer imaging, which, currently, is the best we have achieved in regard to a PD biomarker, does only assess the integrity of the dopaminergic pathways in the striatum and, maybe, although it is controversial, the impact of therapy on these dopaminergic pathways (Agarwal and Stoessl, 2012). However, PD and also the APD, are not only disorders of the dopaminergic system, but of several other neurotransmitter systems, which these radiotracers do not visualize.

In conclusion, biomarkers that can identify and monitor the biochemical effect of drugs, also called “theragnostic markers,” would greatly benefit the search for disease-modifying therapies as well as could be employed usefully as surrogate markers in clinical trials.

### The patho-etiological issue

The ultimate cause of Parkinsonian disorders remains unknown, despite an abundance of theories. Most research has been directed toward the elucidation of the etiology of PD. The vast majority of PD cases are sporadic but approximately 5–10% are genetic. A combination of both environmental and genetic factors is thought to underlie the pathological processes. Considerable evidence implicates oxidative stress in the degeneration of dopaminergic neurons, through deficiencies in the major antioxidant systems, and not only in the brain, but also in the periphery (Jenner, 1991; Kikuchi et al., 2002). Closely linked to oxidative stress is mitochondrial dysfunction (Lin and Beal, 2006). Several hereditary forms of Parkinsonism are caused by mutations in genes related to mitochondria, such as PINK1 and PARK2 (Mortiboys et al., 2008; Gegg et al., 2009). Environmental toxins such as rotenone and paraquat, which can disturb mitochondrial function, are positively associated with PD (Tanner et al., 2011). Alpha-synuclein, a major component of Lewy bodies, inhibits the mitochondrial complex I (Devi et al., 2008) and may cause impaired protein degradation and accumulation of abnormal proteins by disturbing the two major systems which remove damaged proteins: (1) the ubiquitin–proteasome pathway; and (2) the autophagy–lysosome pathway. Transcription abnormalities caused by alpha-synuclein may disturb metabolic pathways (Desplats et al., 2012). Abnormal inflammation in the central nervous system, with activated microglia and massive astrogliosis with increased levels of proinflammatory cytokines (tumor necrosis factor – TNF-α, interleukins), has been found in the CSF in PD; these proinflammatory compounds may promote apoptosis and neuronal death (Hirsch et al., 2003, 2012) and have been suspected to contribute to the development of PD (Czlonkowska et al., 2002) and PSP (Litvan, 2003). Supporting this theory, it has been shown that use of non-steroidal anti-inflammatory drugs (NSAIDs), particularly ibuprofen, was associated with a lower risk for PD (Chen et al., 2003; Gao et al., 2011). It is not known whether the glial activation is secondary to neuronal death induced by other factors, or if it is the primary cause to neuronal death (Schapira and Jenner, 2011).

The cause of MSA, a synucleinopathy, is not known. As for PD, mitochondrial dysfunction and oxidative stress, genetic predisposition, microglial activation, pesticides, and other environmental toxins have been suggested as putative causes (Hanna et al., 1999; Stefanova et al., 2007; Ahmed et al., 2012). Alpha-synuclein accumulates in the oligodendrocytes but its source is not known, neither why it leads to neuronal death. Presumably, disturbances in the neurotrophic support offered by oligodendroglia to neurons result in their degeneration (Ubhi et al., 2011).

As for the synucleinopathies, the ultimate causes of PSP and CBD are not known. Again, a combination of environment and genetics may start the pathological process resulting in accumulation of hyperphosphorylated tau isoforms with four repeats, oxidative stress, and neurodegeneration. Inflammation may also be involved; using PET, microglia cell activation could be found in the same regions where the PSP pathology is usually located (Gerhard et al., 2006).

The bewildering complexity of the current etiological theories may just confirm that we still do not understand the etiology of Parkinsonism but it could also imply that treatment must also be complex and oriented toward several potential targets at the same time (Lang et al., 2012). The same may apply to biomarkers; it could be preposterous to expect to find a single biomarker covering such a complex disease. A biomarker reflecting the etiology of the disease might offer insights into the pathological mechanism itself, thereby opening the way for potentially successful interventions.

## Challenges in the Development of Biomarkers for Parkinsonian Disorders

Although several promising candidates exist, we still lack a reliable biomarker for Parkinsonian disorders. Some of the obstacles on the road to developing biomarkers will be discussed here.

### Disease heterogeneity

In Parkinsonian disorders in general and PD in particular, considering the heterogeneity of clinical presentations at onset, the variability in clinical progression, the multitude of genetic variants and of possible etiologies, it is conceivable that no single biomarker will ever be sufficient, but that several biomarkers will need to be developed, covering biochemical, imaging, pathological, and clinical aspects of the diseases (Marek et al., 2008).

### Diagnostic uncertainties

In Parkinsonian disorders, the diagnosis still remains clinical. Even in the minority of PD cases which are identified through genetic testing, the time for phenoconversion cannot be assessed in a precise and objective way. Clinical diagnostic criteria are susceptible to subjective interpretation and may change over time, as it has happened to a certain degree with PSP. Ultimately, the diagnostic gold standard remains neuropathological examination that can only be ascertained post mortem. Obviously, this is a serious limitation for all research regarding Parkinsonism.

### Slow rate of neurodegeneration in PD

The neurodegenerative process in PD develops insidiously over many years and the degree of degeneration with associated CSF alterations may be too low to be detected by the current laboratory methods. A consequence of that is the high susceptibility to blood contamination which can have profound influence on CSF analysis results.

### Age impact

As most cases of Parkinsonian disorders occur in people aged 55 years and older, there is a high probability for concomitant disorders including neurodegeneration related to other causes, e.g., AD or cerebrovascular pathology. The impact of high age *per se* and comorbidities associated with it has not been sufficiently investigated and more needs to be done in that respect.

### Methodological uncertainties

It is not always clear which kind of measurement is most appropriate and which compounds are the best to explore, making comparisons between studies sometimes difficult. Several of the proteins associated with neurodegeneration are suspected to be aggregation-prone and may exist in different forms, e.g., phosphorylated or unphosphorylated or have different post-translational conformations. Should oligomers or polymers, the mother substance or its metabolites be investigated?

### Blood contamination

While 80% of all proteins in the CSF derive from blood, only 20% are brain-derived (Reiber, 2001). The protein concentration in the blood is much higher than in the CSF, due to the brain-blood barrier which isolates the CSF space. Proteins such as alpha-synuclein are also present in the blood, in erythrocytes, and in thrombocytes. Even minor blood contamination may profoundly affect the results of CSF analysis. The integrity of the blood-brain barrier is crucial for ascertaining that what is found in the CSF reflects the brain environment and not a blood contamination and therefore results from blood-contaminated CSF should not be used. According to one American group, samples should not contain more than 10 erythrocytes per microliter CSF (Caudle et al., 2010), or 500 erythrocytes per microliter CSF according to an European recommendation (Teunissen et al., 2009).

## Future Perspectives

Some of the approaches which may benefit the quest for biomarkers in Parkinsonian disorders are proposed here.

### Standardization of CSF related procedures

Although there are some guidelines in place for the collection and analysis of CSF, from both Europe and the US (Teunissen et al., 2009; Caudle et al., 2010), there is no uniformly accepted protocol making possible the standardization of CSF related procedures. The creation of such a protocol would increase the quality, compatibility, and comparability of CSF related investigations.

### Investigations in unmedicated patients

The impact of dopaminergic medications on potential markers for Parkinsonian disorders is not sufficiently investigated and most of the patients studied so far had been treated with one or more antiparkinsonian medications at the time for LP. There is a need to investigate CSF from unmedicated patients. Some ongoing studies, such as the Parkinson Progression Marker Initiative will make that possible in PD (Parkinson Progression Marker Initiative, 2011) but similar studies are also needed in APD.

### Investigations in early atypical Parkinsonism

Although early PD has been and is being studied, there is a lack of similar studies in early atypical Parkinsonism. This will have to be addressed as understanding the early disease stages probably holds the key to the development of useful biomarkers and efficacious disease-modifying therapies.

### Investigating patterns of potential biomarkers

Given the difficulties encountered when trying to identify single compounds as biomarkers in Parkinsonism, there may be more feasible to identify patterns of compounds serving as biomarkers. Some illustrations of this concept are presented previously in this review. The nature of these disorders may imply minute modifications in single CSF compounds, impossible to perceive, while patterns of several such modifications might be more prone to detection.

### Longitudinal studies

Most of the available data concerning CSF biomarkers comes from cross-sectional studies. Considering the chronic and insidious nature of Parkinsonian disorders, there is a need for longitudinal studies which alone could examine changes and patterns over longer time periods.

### Grouping diagnoses

Grouping diagnoses together, such as PD contra APD or synucleinopathies contra tauopathies, may facilitate developing biomarkers for these diagnostic groups. These biomarkers would be limited and not able to distinguish between single diagnostic entities, but they could be useful in particular circumstances.

### Increased generalizability

All biomarker studies come from highly selected patient populations recruited via movement disorder clinics. In the future it will be necessary to investigate a more heterogeneous Parkinsonian population.

## Conclusion: Clinical Applications of Biomarkers

Reliable biomarkers could be of great use in the development of disease-modifying therapies and in the management of Parkinsonian disorders, once a disease-modifying therapy is developed, by:

(1) Indicating promising therapeutic approaches derived from a patho-etiologic understanding of the disease;
(2) Translating results of drug tests in animals to human populations;
(3) Enriching study populations by identifying patients at risk for a disease;
(4) Determining disease onset at an early stage, hopefully even before the emergence of symptoms;
(5) Stratifying populations according to estimated disease progression, anticipated complications, expected therapy benefits, and potential risks;
(6) Measuring the effects of a therapy on the disease process and on disease progress;
(7) Determining when a therapeutic intervention can be discontinued;
(8) Simplifying the drug regulatory process.

Considering their high positive potential in the management of Parkinsonian disorders, the quest for biomarkers for these diseases must continue unabated.

## Conflict of Interest Statement

The authors declare that the research was conducted in the absence of any commercial or financial relationships that could be construed as a potential conflict of interest.

## Footnotes

^1^http://www.ninds.nih.gov/disorders/clinical_trials/-NCT01280123.htm

^2^http://clinicaltrials.gov/ct2/show/NCT01280123
